# Evaluation of the Clinical Usefulness of a Bioartificial Urethral Substitute Generated by Tissue Engineering in Laboratory Rabbits: Protocol for an In Vivo Study

**DOI:** 10.2196/93091

**Published:** 2026-10-20

**Authors:** David Sánchez-Porras, Fernando Campos, Jesús Chato-Astrain, Miguel Alaminos, M Carmen Sánchez-Quevedo, Ricardo Fernández-Valadés

**Affiliations:** 1 Department of Histology, Tissue Engineering Group School of Medicine Universidad de Granada Granada Spain; 2 Instituto de Investigación Biosanitaria ibs.GRANADA Granada Spain; 3 Division of Pediatric Surgery Hospital Universitario Virgen de las Nieves Granada Spain

**Keywords:** tissue engineering, bioartificial urethra, preclinical assessment, animal experimentation, regenerative medicine, urethral reconstruction

## Abstract

**Background:**

Surgical management of diseases compromising the integrity of the human urethra remains a major clinical challenge, and congenital conditions, such as hypospadias and epispadias, and acquired diseases, such as trauma, tumors, and strictures, are difficult to treat, highlighting the need for novel regenerative approaches capable of restoring urethral architecture and function. Recent advances in tissue engineering have allowed the generation of bioartificial tissues, including several models of artificial urethra generated with different types of cells and biomaterials. Although potentially useful, these models cannot reproduce the multilayered native human urethra structure. One biomaterial showing excellent biocompatibility for use in tissue engineering is fibrin-agarose hydrogel, which has been used with promising results in patients with severe alterations of the cornea, skin, and oral mucosa, suggesting its potential use for multilayered urethral substitutes.

**Objective:**

This study aims to evaluate the biosafety and efficacy of a multilayered artificial urethra generated by tissue engineering using fibrin-agarose biomaterials in laboratory rabbits to determine its clinical potential as an advanced therapy medicinal product.

**Methods:**

Five study groups will be established, including a positive control group of rabbits with a native (ie, normal) urethra, a negative control group of rabbits in which the ventral side of the urethra will be surgically sectioned and left unrepaired, a gold standard group in which the ventral side of the urethra will be surgically sectioned and repaired with an oral mucosa autograft, an acellular group in which the urethral defect will be repaired using an acellular multilayered fibrin-agarose biomaterial, and a cellular group of rabbits in which a multilayered cellular bioartificial urethra will be used to repair the urethral defect. The cellular group will allow us to assess the usefulness of a biomimetic urethral substitute generated by tissue engineering, as demonstrated in previously published models of bioartificial tissues, whereas the acellular group will allow us to determine whether the effects observed for the artificial urethra are attributable to the biomaterial itself, which could act as a physical barrier, or to the action of the cells. In all cases, animals will be observed for 3 months, and variables related to biosafety, functionality, and the histological structure of the urethral grafts will be determined.

**Results:**

This study was approved by the ethics committee for animal research (Junta de Andalucía; 17/12/2024/178) on December 20, 2024. The in vivo study began in February 2025 and is expected to be completed by December 2026. The study was funded in January 2023, and data collection is expected to be completed by December 2026. The results are expected to be published in 2026 and 2027.

**Conclusions:**

The present preclinical study will contribute to determining the potential usefulness of these bioartificial tissues as novel advanced therapy medicinal products for clinical use.

**International Registered Report Identifier (IRRID):**

DERR1-10.2196/93091

## Introduction

The clinical management of urethral disorders is very complex. Both congenital conditions, such as hypospadias or epispadias, and acquired disorders severely affecting the urethra, including trauma, tumors, and strictures, pose a major therapeutic challenge in urology [1-3]. In these cases, complex surgical interventions are needed, and results are highly heterogeneous [4]. In fact, recent studies have demonstrated that up to 90% of urethral strictures tend to recur within 48 months after treatment and require additional interventions [5].

One of the most commonly described therapeutic options, especially in cases of severe stricture, is the use of skin [6] and oral mucosa autografts, which can be applied as onlay graft implants [7]. Although this technique can be considered the gold standard treatment in specific cases, it has been shown that oral mucosa autografts are associated with high rates of disease recurrence, meatal stenosis, and donor site morbidity [8-10], and results remain highly variable [3]. In addition, oral mucosa autografts have important limitations, including donor site morbidity, restricted size, and limited functional capability [11]. For these reasons, novel therapeutic alternatives achieving improved results are needed.

In this regard, advances in tissue engineering have allowed the efficient generation of bioartificial tissues and organs in the laboratory by combining cells, biocompatible biomaterials, and growth factors [12], and potentially useful models of different human organs have been described to date [4,13]. In the case of the urethra, interesting bioartificial models have been developed using different types of biomaterials, including collagen, polylactic acid, and synthetic scaffolds produced by 3D printing, among others [14]. However, there is no consensus on the optimal biomaterials that allow the efficient generation of urethral substitutes, and further research in this field is needed [14]. Among the available biomaterials, fibrin-agarose hydrogel emerges as a promising biomaterial for use in urethral tissue engineering. This natural scaffold was authorized by the Spanish Medicines Agency and has shown high biocompatibility and good clinical results in patients with severe corneal, skin, and oral mucosa diseases [15], supporting its use in urethral reconstruction.

In addition, the human urethra is a complex organ consisting of 3 main layers: the urethral mucosa (UM), the spongy layer (SP), and the tunica albuginea (AL), and most of the previously published models of artificial urethra fail to reproduce all the layers of this organ. As reproducing the structure and function of native tissues is a requirement for tissue-engineered tissues to be clinically useful [16], further advances in biofabrication are required to obtain a fully biomimetic urethral substitute. In this context, we recently described a full-thickness bioartificial urethra generated by tissue engineering, consisting of the 3 main layers of this organ [17]. Although this urethral substitute may have therapeutic potential, its in vivo efficacy needs to be determined in laboratory animals to assess its safety and functionality.

Furthermore, it has been described that the use of mesenchymal stem cells (MSCs) with high differentiation potential may allow the generation of bioartificial tissues by tissue engineering, without the need to obtain biopsies of the target organ [18], and promising substitutes of the human skin, cornea, and ureter have been developed using MSCs [19]. Compared with orthotypical cells obtained from the urethra, MSC typically show a high proliferation rate and enhanced differentiation potential, and their applications in regenerative medicine have expanded considerably in recent years [20]. In addition, MSC have intrinsic immunomodulatory properties, which could contribute to reducing inflammation and controlling in vivo cell-cell responses [21]. Although a full-thickness urethral substitute has not yet been generated using MSC, previous studies support the differentiation potential of these cells toward the urethral lineage and their applicability to the generation of bioartificial urethral tissues by tissue engineering without the need to obtain urethral biopsies [22,23]. By contrast, some reports have shown that acellular scaffolds could support urethral regeneration, suggesting that the use of cells may not be strictly required in all cases [24]. In fact, acellular scaffolds have been successfully used for the regeneration of several organs and tissues, including skeletal muscle [25] and cartilage [26], among others.

In this work, we describe a preclinical study protocol whose main objective is to determine the biosafety and potential in vivo usefulness of a cellular multilayered bioartificial urethra generated by tissue engineering using MSC (cellular multilayered urethral substitute graft [BIOUR]) in laboratory animals, as a key step in the clinical translation of this product as an advanced therapy medicinal product (ATMP). Secondary objectives include comparing the product with the current gold standard treatment and investigating the role of the MSC in the urethral substitute. To achieve these objectives, the New Zealand laboratory rabbit was selected as the in vivo experimental model. This animal is a widely recognized, highly reliable preclinical model for urethral research due to the anatomical and functional characteristics of this species. Its urethral length allows the surgical implantation of biomaterials and bioengineered tissues, enabling the creation of urethral defects. For these reasons, laboratory rabbits have previously been used in studies of urethral pathology, demonstrating their suitability for evaluating urethral tissue regeneration, graft integration, and functional outcomes [27-29].

Our hypothesis is that novel fibrin-agarose urethral substitutes will show in vivo therapeutic potential, and animals treated with the BIOUR substitute will show favorable regeneration results without adverse effects.

## Methods

### Generation of Cellular and Acellular Multilayered Bioartificial Urethras by Tissue Engineering

Generation of cellular and acellular multilayered bioartificial urethras by tissue engineering occurred as follows. First, we will fabricate a bioartificial substitute of the UM, the SP, and the AL using nanostructured fibrin and fibrin-agarose biomaterials, following previously published protocols [17], using rabbit adipose-derived MSCs and fibroblasts. In brief, UM and AL substitutes will be generated using fibrin-agarose biomaterials, while fibrin will be used for SP substitutes. In the case of UM, fibroblasts will be immersed within the hydrogels, and MSCs will be seeded on top, whereas for SP and AL, MSCs will be immersed within the stroma. In all cases, cell differentiation into specific cell types (urothelial, vascular, and smooth muscle cells) will be induced by applying specific induction media and cell culture conditions, as previously reported [30,31]. After 21 days of induction, the UM, SP, and AL layers will be fused into a multilayered structure by applying nanostructuration methods based on plastic compression [17,32]. The final product containing the 3 layers will be considered a cellular multilayered bioartificial urethra generated by tissue engineering using MSC (BIOUR).

In addition, acellular scaffolds will be generated by tissue engineering for use in the group of animals receiving an acellular multilayered bioartificial urethra generated by tissue engineering (UREG group). In this case, we will first generate the UM, SP, and AL layer substitutes using the same biomaterial and protocols described for BIOUR, but no cells will be added to the mixture. The final acellular product will be generated by applying the same nanostructuration methods described for the cellular substitute to obtain a multilayered acellular substitute. The acellular multilayered bioartificial urethra will be identical to the cellular product, except for the presence of cells. All tissue-generated products will be kept in a cell incubator at 37 °C with 5% carbon dioxide (CO_2_) for 24 to 48 hours until their implantation in laboratory animals.

### Animal Experimentation

Evaluation of the urethral substitutes described in this work will be performed on adult male New Zealand laboratory rabbits aged 6 months and weighing approximately 4 kg. We will use only healthy, nongenetically modified animals. As a preliminary exploratory study, a formal sample size calculation was not performed because reliable estimates of effect size and variance are not available for the bioengineered tissues generated in the present work. Instead, 5 animals will be randomly assigned to each group, as no inclusion or exclusion criteria have been established (total 25 animals). This sample size was selected based on previous preliminary studies in rabbits that also used small groups of animals [33], in which these sample sizes allowed the efficient detection of differences among groups regarding graft integration, tissue regeneration, urethral function, and other outcome analyses. According to our experience with other types of bioartificial tissues evaluated for the first time [34], the proposed study is expected to identify relatively large biological effects associated with successful vs unsuccessful urethral regeneration, rather than subtle differences between treatments, and it is well known that small sample sizes are acceptable when biological variability is expected to be low and effect sizes are expected to be large [35]. In addition, the use of highly standardized surgical procedures, homogeneous animal characteristics (same species, sex, age, and weight range), and controlled experimental conditions is expected to reduce biological variability, thereby increasing the likelihood of detecting relevant treatment-related effects despite the limited sample size. The information obtained from this study will provide the preliminary estimates of variability and effect size required for future power calculations and the design of larger confirmatory studies. Furthermore, the use of a low sample size is consistent with the 3Rs (replacement, reduction, and refinement) principle, suggesting that animal numbers should be minimized as much as possible [36]. Each animal will be assigned a unique identification number and randomly allocated to the experimental groups using a computer-generated randomization sequence generated with the online tool Randomizer.org [37]. Group allocation will be performed by a researcher from the animal facility who will not be involved in the surgical procedures or outcome assessment. Group assignment will not be blinded to the surgeons performing the implantation procedures, as they must know which treatment is to be applied to each animal. However, researchers in charge of the functional and histological analyses will be blinded to group allocation.

### Study Groups and Experimental Procedures

The following 5 study groups will be established.

The positive control group (P-CTR), comprising animals in which the urethra will not receive any intervention. They will be considered to have a native, normal urethra.

The negative control group (N-CTR), comprising animals in which the ventral side of the urethra will be surgically sectioned following the urethral axis. This defect will be sutured and repaired without using any tissue grafts (primary closure).

The oral mucosa autograft (AUTO) group, comprising animals in which the ventral side of the urethra will be surgically sectioned following the urethral axis. This defect will be repaired by grafting a fragment of autologous oral mucosa obtained from the inner cheek of the animals, following procedures typically used in human surgery.

The acellular multilayered urethral substitute graft (UREG) group, comprising animals in which the ventral side of the urethra will be surgically sectioned following the urethral axis. This defect will be repaired by grafting an acellular multilayered urethral substitute generated by tissue engineering as described above.

The BIOUR group, comprising animals in which the ventral side of the urethra will be surgically sectioned following the urethral axis. This defect will be repaired by grafting a cellular multilayered bioartificial urethra generated by tissue engineering using MSC (BIOUR), as described above.

Animals subjected to surgery (N-CTR, AUTO, UREG, and BIOUR) will be deeply anesthetized using an intramuscular injection of ketamine (25 mg/kg) and xylazine (5 mg/kg) before surgery, and postsurgical analgesia will be administered in the drinking water (magnesium metamizole, 1.6 mg/mL) for 48 hours to ensure animal welfare. Once anesthetized, the penile urethra will be catheterized using a vaselinated silicone tube, and the skin will be excised approximately 5 mm below the glans and retracted to expose the penile corpus and the urethra. Then, the ventral surface of the penile urethra will be incised along the longitudinal (major) axis of the urethra, approximately at the midportion of the penile urethra, using a sharp-tipped surgical knife or scissors. The size of this defect will be approximately 5 mm. Although this defect size is not considered critical, its size allows the implantation of a bioartificial tissue suitable for evaluating early healing and integration without the morbidity associated with larger defects frequently used in rabbit experimentation. In the AUTO group, an onlay graft obtained from the oral mucosa of the same animal will be grafted to repair the defect using absorbable polyglactin 910 (Vicryl 7/0) sutures (Ethicon Inc). In the UREG group, this defect will be repaired with the acellular multilayered urethral substitute, whereas in the BIOUR group, the defect will be repaired with the BIOUR cellular substitute, using the same suture material. In all cases, the retracted penile skin will be reintegrated to its original place and fixed with the same suture material. The urethral catheter will remain in place for 3 days after surgery to ensure adequate urinary drainage during the early postoperative period. The surgical procedure is shown in Figure 1. All surgical interventions will be performed only once, after which the animals will be allowed to heal. All animals will be euthanized and evaluated after 3 months of follow-up, in accordance with previously published protocols evaluating different types of implants in the rabbit urethra that demonstrated that this time period is sufficient to assess urethral regeneration and functional outcomes [38,39].

**Figure 1:**
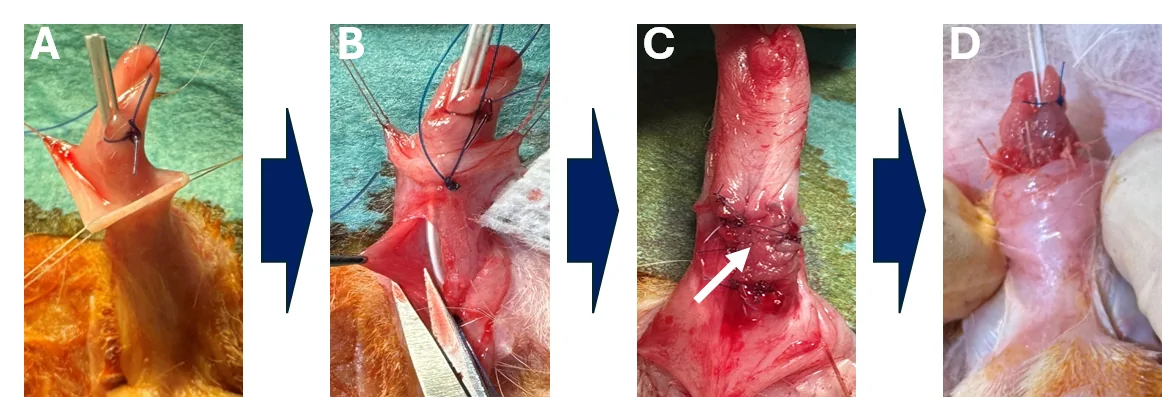
Surgical images illustrating different stages of the surgical procedure performed on animals in the oral mucosa autograft (AUTO), acellular multilayered urethral substitute graft (UREG), and cellular multilayered urethral substitute graft (BIOUR) groups. (A) Catheterization of the penile urethra; (B) exposure of the penile urethra and longitudinal incision; (C) implantation of a tissue graft (white arrow) at the defect site, secured with absorbable sutures; and (D) final appearance after repositioning the penile skin to its original location and fixation with sutures.

### Follow-Up and Study Variables

Animals will be evaluated daily, and the final results will be assessed after 3 months of follow-up, at which point animals will be euthanized with Eutanax 200 solution (Fatro Ibérica) under general anesthesia for histological analysis of the urethral tissues. Each animal will be analyzed individually (experimental unit: individual animal). All animals (n=5 per group) will be included in the analysis, and no exclusions will be made. To minimize potential confounders, animals will be randomly allocated to each cage at the animal facility.

In each study group, the following variables will be analyzed. First, primary outcome variables will be analyzed. The feasibility of the surgical procedure will be assessed by surgeons in charge of the surgical procedures using a Likert-like scale as follows: 1=“the procedure is not feasible and cannot be achieved,” 2=“the procedure is slightly feasible,” 3=“the procedure is moderately feasible,” 4=“the procedure is very feasible,” and 5=“the procedure is completely feasible and can be achieved without any problem.” This scoring system was designed ad hoc for the present work and was adapted from a previously published scale developed by our group to assess the feasibility of grafting a bioengineered palate mucosa in patients with cleft palate [34]. To minimize subjective bias, 3 independent surgeons will evaluate this parameter. In cases of disagreement, the median score will be used for the final analysis. The biosafety of the grafted tissues and tolerance of the animals to the procedure will also be assessed. Any surgical or postsurgical mortality, complications, or side effects will be recorded for each animal, including bleeding, necrosis, infections, and dehiscence of the sutures.

Second, secondary outcome variables will be analyzed. Difficulty voiding will be assessed by analyzing the capability of each animal to urinate without complications. The shape of the urine stream, the presence of incontinence, and any abnormal urination will be assessed in each group. For this purpose, we will analyze the urine stains left by the animals on absorbent pads placed under the cages. In addition, the bladder of each animal will be filled with dye-containing water, and the morphology and direction of the urinary stream will be visually inspected after the established follow-up period and after the animals have been euthanized. Anatomical alterations will be assessed by ocular inspection to determine whether the penile urethra of each animal develops any morphological alterations derived from the surgical procedure (eg, megaurethra, fistulization, and stenosis). Histological parameters will be assessed after the follow-up period. The penis of each animal will be carefully dissected, fixed in formalin, and embedded in paraffin, and 3 µm thick tissue sections will be obtained using a microtome. For histological assessment, sections will be dewaxed and stained with hematoxylin-eosin. To identify specific components of the tissue extracellular matrix, sections will be subjected to histochemistry for collagen, elastic fibers, proteoglycans, and glycoproteins, as previously described [40]. The histochemical signal will be quantified in each study group using the histological quantification system ImageJ (National Institutes of Health), following previously published protocols [40]. In brief, histological images will be obtained using similar conditions for all study groups, and automatic signal quantification will be performed to determine the average staining intensity in the areas of interest. Specific markers will be detected by immunohistochemistry for cytokeratins (CK1, CK7, CK8, and CK19), involucrin, uroplakin, versican, biglycan, decorin, and other proteins involved in urethral differentiation and function. Results of the immunohistochemical analyses will be semiquantitatively evaluated using a previously established scoring system and classified as strong positive staining signal (+++), positive staining signal (++), mildly positive staining signal (+), or negative (−) [40,41]. In each study group, potential immune-related effects of the treatment will be histologically evaluated, and any signs of inflammatory reaction, necrosis, fibrosis, hemorrhage, or other side effects will be evaluated.

The overall flow of the study is shown in Figure 2.

For the quantitative parameters, a statistical analysis will be performed to compare the results obtained among the different groups. First, the normality of each distribution will be determined using Shapiro-Wilk tests, and homogeneity of variances will be evaluated using the Levene test. Then, multigroup comparisons will be performed using 1‑way ANOVA (if distributions meet parametric criteria) or the Kruskal-Wallis test (if criteria are not met). Variables showing statistical differences will subsequently be analyzed using post hoc pairwise comparisons between 2 specific study groups using the Student *t* test (parametric data) or the Mann-Whitney test (nonparametric data). A Bonferroni-adjusted *P* value will be established to correct for multiple comparison errors. These analyses will be carried out using Real Statistics Resource Pack software, from Purdue University, West Lafayette, IN, United States [42], as previously reported [15,17]. Any unexpected animal deaths, missing data, and protocol deviations will be documented. Animals that die before completion of the experimental procedures or from causes unrelated to the study intervention will be excluded from end point–specific analyses, and the reason for exclusion will be clearly stated. No imputation of missing data will be performed.

**Figure 2:**
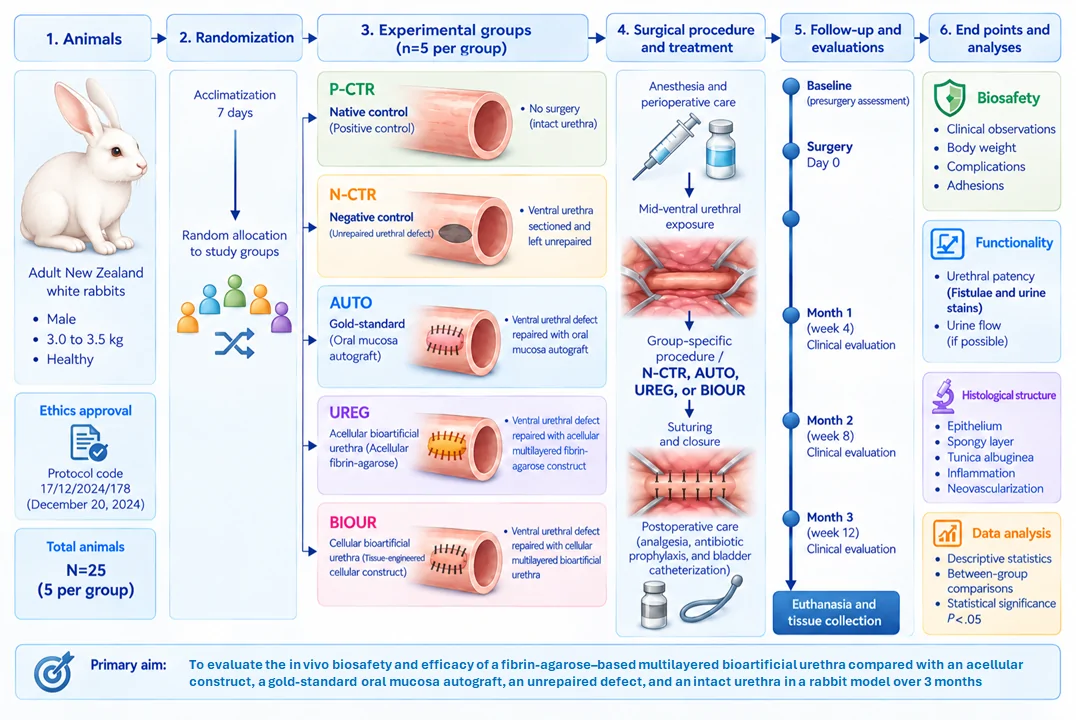
Study workflow showing animal allocation, randomization into 5 experimental groups, surgical intervention, 3-month follow-up, and end point analyses evaluating biosafety, functionality, histology, and treatment efficacy.

### Ethical Considerations

Animal studies will follow European Union and Spanish guidelines for ethical animal care (EU Directive 63/2010; RD 53/2013). All animals will be housed in individual cages measuring approximately 80×60×45 cm at the animal facility of the Experimental Unit of the University Hospital Virgen de las Nieves and ibs.GRANADA Health Research Institute in Granada (Spain), where all surgical procedures will be performed, in a temperature-controlled room (21±1 °C) under a 12-hour light-dark cycle, with free access to water and standard rabbit chow. Environmental enrichment will be provided using hay and plastic shelters. On arrival, animals will be acclimatized to the housing conditions for 7 days, following standardized protocols of the Experimental Unit. Humane end points were predefined, and animals will be euthanized under general anesthesia if severe symptoms are detected, such as persistent anorexia, distress, lethargy, signs of pain or suffering, abnormal behavior, severe infection, or other conditions detected by the attending veterinarian of the Experimental Unit that could compromise animal welfare. Animal experimentation will be reported following the ARRIVE (Animal Research: Reporting of In Vivo Experiments) Guidelines 2.0 for reporting animal research [43].

This study was reviewed and approved by the Institutional Ethics Committee, Comité Coordinador de Ética de la Investigación Biomédica de Andalucía (CCEIBA; 0268-N-23) on April 13, 2023, and by the Consejería de Agricultura, Pesca, Agua y Desarrollo Rural (Junta de Andalucía; 17/12/2024/178) on December 20, 2024. Animal welfare will be ensured at all times.

## Results

This study received funding in January 2023, was authorized by the ethics committee in 2023 and 2024 and is currently in progress. The in vivo study in laboratory animals began in February 2025 and is expected to be completed by December 2026. Researchers have already collected some preliminary results related to the use of an acellular urethral substitute in the UREG group, suggesting that this strategy may be beneficial for urethral regeneration.

Data collection and animal experimentation are expected to be completed by December 2026. No protocol deviations have been introduced, and the final results are expected to be published in the second half of 2026 and in 2027. The planned timeline of the project is shown in Figure 3.

**Figure 3:**
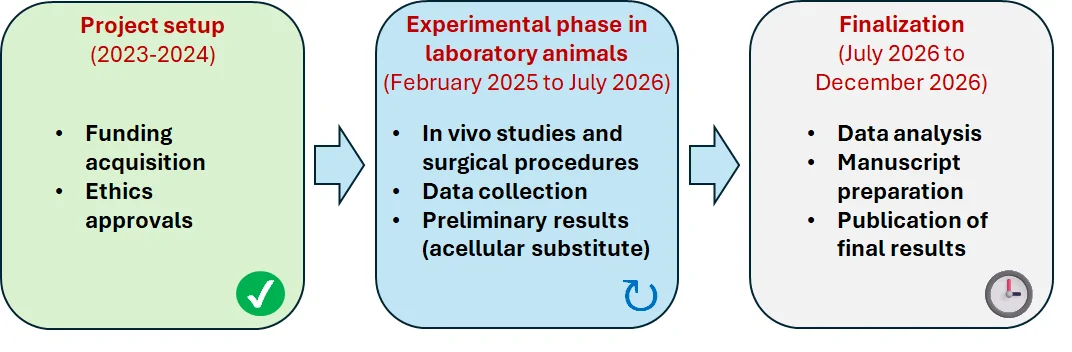
Planned timeline and activities of the project.

## Discussion

In the present work, we describe a study protocol to evaluate a bioartificial urethra generated by tissue engineering. Although significant advances have been achieved in the field, the treatment of severe conditions affecting the human urethra remains a major clinical challenge requiring further research [22]. Several models of bioartificial urethras have been reported to date using different types of biomaterials, such as collagen, silk, decellularized tissues, and synthetic materials [23,44,45]. However, none of these models is able to fully reproduce the structure of the native urethra, especially its multilayered structure, which warrants further research in this area. In addition, the use of tissue-engineered urethras in animal models is typically associated with a high rate of complications, especially strictures and fistulae, which have significantly hindered clinical translation [46].

In comparison with other types of biomaterials, fibrin-agarose hydrogel has demonstrated high biocompatibility and good tolerance when grafted in laboratory animals [17,19,33] and has allowed the generation of multilayered structures by using nanostructuration methods [32]. Furthermore, fibrin-agarose biomaterial was authorized by the Spanish Medicines Agency for the clinical treatment of patients with severe burns, with promising biosafety and biofunctionality results [15]. By using this biomaterial, the present project proposal aims to generate a potential multilayered urethral substitute that will be evaluated in the urethra of laboratory animals, where this material has not been tested to date. On the basis of our previous findings, we hypothesize that this biomaterial will also be well tolerated when applied to the rabbit urethra.

However, it is unknown whether the use of acellular biomaterials in the UREG group may or may not be associated with optimal results. In this regard, it has been demonstrated that urethral implantation of acellular biomaterials, such as collagen or fibroin, can lead to scaffold degradation and fibrosis, with the formation of cicatricial tissue at the implantation site [47-49]. Therefore, we have established a study group in which a cellular urethral substitute will be grafted (BIOUR), as it has been demonstrated that the presence of cells in the scaffold can inhibit urethral fibrosis and favor tissue regeneration [50]. Although there is no clear evidence supporting their positive effect in urethral tissue engineering, the immunomodulatory properties of MSCs might contribute to preventing excessive inflammation and fibrosis [21]. In fact, it has been shown that MSC-derived exosomes can reduce urethral scarring and stricture formation [51], and we hypothesize that the cellular bioartificial urethras generated in the present work may have potential in vivo usefulness in laboratory rabbits. Furthermore, the generation of a multilayered substitute of this organ is very complex and requires the use of advanced biofabrication methods and techniques, such as nanostructuration, which we previously used to generate multilayered organs by tissue engineering [32], including the human urethra [17].

The present project will determine if this novel urethral substitute may have potential clinical usefulness in patients with severe urethral conditions. According to the guidelines of the European Medicines Agency (EMA), a thorough preclinical evaluation demonstrating in vivo biosafety and functionality is required for novel products intended for clinical use [52]. Results from the present study will be used to determine the translational potential of this product in patients with severe urethral conditions and to evaluate the possibility of generating the artificial urethral substitute as a novel ATMP. However, successful preclinical outcomes represent only an initial step toward clinical translation. If the product meets the biosafety criteria established by the EMA and demonstrates potential in vivo usefulness in urethral regeneration, clinical translation would require overcoming several important challenges, including the standardization and scalability of the manufacturing process, the establishment of robust quality control procedures, batch-to-batch reproducibility, long-term product stability, and the economic feasibility of large-scale production, including all the quality controls that such production requires [15]. In addition, regulatory approval would require comprehensive characterization of the cellular and biomaterial components of the bioartificial tissues, demonstration of product consistency, and comprehensive preclinical data addressing safety, biodistribution, immunogenicity, and long-term functionality. If these criteria are successfully met, bioartificial urethras would need to be generated in a Good Manufacturing Practice (GMP) facility as novel ATMPs, which would then need to be evaluated in patients with urethral conditions in the context of an advanced therapy clinical trial that would also need to be authorized by the EMA or the corresponding national medicines agency [52]. The present work is a critical step in the ATMP translational pathway of these bioartificial urethras, as it aims to establish robust in vivo evidence of biosafety and functionality for future clinical applications.

Although previous studies carried out by our research group demonstrated that bioartificial tissues generated with allogeneic MSCs are poorly immunogenic, due to the immunoprivileged nature of these cells [19], caution remains necessary when using the bioartificial urethra generated in the present study. Indeed, allogeneic MSC could potentially induce host immune responses characterized by inflammatory or immune cell infiltration, and these effects should therefore be carefully monitored [53]. For these reasons, we will evaluate immune-related effects of the treatment using histological analyses capable of identifying any signs of inflammation, inflammatory infiltration, or rejection due to the use of allogeneic cells. These analyses will allow us to distinguish normal regenerative processes associated with graft integration from potential adverse reactions, thereby providing additional information regarding the biocompatibility and safety of the implanted artificial tissues.

The selection of New Zealand laboratory rabbits as an in vivo model for experimental urethral research was based on previous studies demonstrating that they are a well-established experimental model [27-29]. Laboratory rabbits remain a valuable preclinical model because of their urethral size, relatively low cost, and proven utility in urethral research. However, results should be interpreted with caution due to the important anatomical and physiological differences between humans and rabbits. Moreover, the wound healing response of these animals may not completely replicate that observed in humans. These limitations may limit the direct translation of the results to clinical practice.

This project has several limitations, including the different physiology of human and animal tissues and organs, the limited sample size, and the lack of objective methods for evaluating voiding function. Future studies should address these limitations by incorporating comprehensive functional evaluations, including uroflowmetry, imaging, and urodynamic pressure measurements [54]. These analyses will be essential to determine both the short- and long-term functional performance of the engineered urethral substitutes used in the present work and to strengthen the relevance and translational significance of our findings. However, 1 advantage of the project is the inclusion of positive and negative study groups, along with a group in which the gold standard treatment—oral mucosa autograft—will be applied. In addition, the project will evaluate not only the BIOUR product but also the acellular biomaterial.

In conclusion, the protocol described in the present work is for one of the first in vivo studies to evaluate a multilayered urethral substitute generated by tissue engineering. Results from the study will allow us to determine the potential usefulness of this product as a novel ATMP for use in patients with severe urethral damage.

## Appendix

Multimedia Appendix 1 ARRIVE guidelines checklist.

## Abbreviations

AL: tunica albuginea
ARRIVE: Animal Research: Reporting of In Vivo Experiments
ATMP: advanced therapy medicinal product
AUTO: oral mucosa autograft group
BIOUR: cellular multilayered urethral substitute graft
CO2: carbon dioxide
EMA: European Medicines Agency
GMP: Good Manufacturing Practice
MSC: mesenchymal stem cell
N-CTR: negative control group
P-CTR: positive control group
SP: spongy layer
UM: urethral mucosa
UREG: acellular multilayered urethral substitute graft

## Data Availability

Data generated during this study will be deposited in the European public repository Zenodo and will be freely available without restrictions, according to the Creative Commons Attribution 4.0 International License.
